# Climate Change and Insects

**DOI:** 10.3390/insects14080678

**Published:** 2023-07-31

**Authors:** Michael Eickermann, Jürgen Junk, Carmelo Rapisarda

**Affiliations:** 1 Luxembourg Institute of Science and Technology (LIST), Environmental Research and Innovation Department (ERIN), 41 Rue du Brill, L-4422 Belvaux, Luxembourg; juergen.junk@list.lu; 2 Dipartimento di Agricoltura, Alimentazione e Ambiente (Di3A), Università degli Studi di Catania, Via Santa Sofia 100, I-95123 Catania, Italy; carmelo.rapisarda@unict.it

Climate change (CC) poses one of the foremost challenges for humanity in the 21st century. Based on numerical climate projections, the global near-surface temperature is expected to rise by 1.6 °C to 2.4 °C by 2050 across Central Europe [1]. Insects are the focus of CC impact studies due to their role as important pests in agriculture and forestry [2,3] as well as their role in contributing to different ecosystem services [4]. Most research still focuses on the impact of CC on the distribution and dynamics of single insect species (e.g., [5]), while studies investigating CC’s influences on multi-trophic interactions or species communities in single habitats or ecosystems are missing.

This Special Issue claims to encompass multiple facets of how insects are expected to respond to global and regional environmental changes. The intention is to include various species from diverse orders in order to demonstrate the impact of climate change on a wide diversity of insects. Therefore, this Special Issue deals with insect species from Coleoptera [6], Diptera [7], Hymenoptera [8,9], Lepidoptera [10,11,12], Odonata [13], and Thysanoptera [14]. The regions covered by the articles range from local (e.g., southeastern Sweden) to larger geographical areas (e.g., the Middle East) and the global scale.

In this editorial, we want to highlight articles that reveal recent discoveries in fundamental and applied research on insects under global warming. The articles published here can be separated into two categories, dealing with the effects of CC on (a) insects’ life cycle and (b) potential geographical distribution patterns, including insights into the population dynamics of insects. It must be underlined that some articles discuss the economic impacts of CC on insect distribution, especially under plant protection aspects [14] and—quite specifically—in apiculture [10]. Aspects of species conservation are also addressed in at least three articles [6,7,13], as well as the energetic costs for species development cycles [8].

Most studies in this Special Issue utilize numerical climate projections derived from global and/or regional climate models. Data mostly originate from the Coupled Model Intercomparison Project Phase 5 (CMIP5) available at the WorldClim portal (www.worldclim.org), covering different emission scenarios with a main focus on RCP4.5 and RCP8.5. To tackle the uncertainties associated with climate projections, a substantial portion of the papers rely on a multi-model ensemble approach by incorporating outputs from various climate models. Some studies rely on long-running and multi-sited monitoring activities, while others focus on potential distribution patterns based on sophisticated modeling approaches like Biomod2 or the Maximum Entropy (MaxEnt) Model. The last one has become most popular during the last twenty years, offering a precise predictive performance with a high level of accuracy [10]. Therefore, it is unsurprising that five articles in this issue rely on it.

Most papers in this Special Issue encompass two types of Representative Concentration Pathways (RCPs), mostly RCP2.6, RCP4.5, and/or RCP8.5. Only the articles by Gao et al. [9] and Wang et al. [14] included three emission scenarios to cover a broad range of possible future climate realizations. Concerning the timespans covered by the projections, the authors focus on two different future timespans: the near future (2041–2060) and the far future (2071–2100).

Monitoring and observations of insect activity are fundamental to detecting changes in species composition in a dedicated area as well as shifts in their activity pattern. Betzholz et al. [11] focused in their article on a rapid distribution shift of range-expanding moths in southeastern Sweden based on a 16-year survey. They measured an increase in species abundance and population growth, followed by a significant growth rate of the population after the first colonization due to increased air temperatures. This study is the only one in this Special Issue that underlines the potential increase in species richness and abundance due to climate change. The authors also discussed the potential effects of shifts in insect communities on the functioning of ecosystems. Compared to the other articles in this issue, their study is not based on climate projections and potential effects [11]. Here, a long-term monitoring dataset proves the effect of rapid warming on insects and underlines the importance of monitoring activities in entomology.

Climate warming has an impact on habitat selection as well as the geographical expansion of insects. The habitats need good conditions to guarantee a full life cycle of an insect generation. Kovac et al. [8] analyzed the environmental conditions (air temperature, solar radiation) at the nest of different *Polistes* (Hymenoptera) species in different areas in Europe. By using adequate body temperature models, the authors identified the energetic demand of the wasps during the breeding season. They underlined the importance of fine-scale microclimate measurements in the insect’s habitats as a crucial necessity. The authors proved clearly that *Polistes* species from warmer climates showed a higher metabolic rate with increased air temperature due to higher costs for basic subsistence and activity. On the contrary, species from cooler habitats could profit from higher air temperatures by potentially longer periods of foraging and a shorter development time for the brood [8].

By investigating potential habitats and distribution ranges based on meteorological variables, CC impact studies can be a useful tool to foster species conservation. In this Special Issue, Fekete et al. [13] analyzed the future distribution of *Cordulegaster* (Odonata) species by weighting different climatic variables. The genus is well-known for its narrow habitat preferences. Two species—*C. bidentata* and *C. heros*—were strongly affected by bioclimatic variables and showed an upward shift toward higher elevations. In addition, the model output by the authors also showed that areas suitable for the *Cordulegaster* species at present could become unfavorable in the future under CC effects.

Like *Cordulegaster* spp., species of the genus *Osypha* (Coleoptera) have high requirements for their habitat, and therefore most of the *Osypha* species are endangered. The study by Liu et al. [6] can provide information for protecting this beetle group in a changing environment focusing on distribution patterns on a global scale. Based on a comprehensive geographic distribution dataset combined with CC projections, warm, stable, and rainy climates like the coastal areas of the USA are most suitable for *Osypha* species that also tend to spread northwards towards higher latitudes. This information will lay the basis for the protection efforts of these relict insects in the future.

While so many insect species are under pressure from environmental effects, insect pests seem to increase their population sizes under CC. Therefore, the future fate of insect species that are dedicated pests in agriculture and forestry is of special interest due to their economic impact [3]. Regnier et al. [12] evaluated the potential impacts of CC on the development time of stem borers (Lepidoptera) in maize in tropical and temperate climates, e.g., *Ostrinia nubilalis*. Based on temperature-dependent mathematical models for all four species, the authors described the beneficial as well as detrimental effects of CC in terms of the high dependency on the optimal temperature for species development. The authors expected, in the short term, an accelerated development for all investigated species, while in the long run, an acceleration, as well as a delay in development, can be expected, depending on the region [12]. Potential effects on population dynamics are discussed. This study clearly underlines that CC impact studies have to include different future timespans to identify significant effects on insects’ lifecycles in a changing environment, potentially leading to changes in their spatio-temporal dynamics. Therefore, adaptation of monitoring as well as developing interventions to manage pests will be necessary in realize practical farming to secure food production

Pathogen-transmitting insects like aphids or whiteflies are, next to herbivores, the focus of many research activities worldwide [15].Wang et al. [14] investigated the effect of CC on the potential distribution of the pest *Franklinella occidentalis* (Thysanoptera) in China. Their results indicated that highly suitable areas for this pest will be widely distributed in 19 provinces of China. The authors discussed in detail potential control strategies, such as prevention methods, to avoid the spread of the pest in new habitats. It is interesting that the authors also mention the use of biopesticides and antagonists as a substitute for synthetic pesticides [14].

Next to pests on crops, pests in livestock management are a dedicated research topic as well. Hosni et al. [10] highlighted the effect of CC on the greater wax moth (*Galleria mellonella*) (Lepidoptera), which is a common pest in beehives and well known by beekeepers. The authors investigated the distribution of this pest under projected CC scenarios and compared the habitat suitability with hotspots of regional apiculture. Next to some European countries, the authors identified the beekeeping industry on the Australian eastern coast and in New Zealand as highly susceptible to wax moth calamities in the future [10].

Antagonists of insect pests, like predators and parasitoids, play an important role in plant protection, especially in greenhouses. However, like their host organisms or prey, they can also be affected by CC. Gao et al. [9] presented a study on *Sclerodermus sichuanensis* (Hymenoptera), an effective parasitoid of larvae and pupae of longicorn beetles. Concentrating on different meteorological variables for CC projections, such as the mean diurnal range and min temperature of the coldest month, the authors analyzed potential distribution areas in China. Gao et al. [9] discussed a significantly extended range of this parasitoid for biological control of pests in forestry under CC effects. Another study in this Special Issue deals with a parasitoid: Soliman et al. [7] investigated the future distribution of *Spogostylum ocyale* (Diptera), a larval ectoparasitoid of solitary bees and an effective pollinator itself. Based on climate projections, the authors showed a progressive decline in the extent of suitable habitats for this species under CC. This study relies on a bundle of different meteorological variables, from annual mean temperature to precipitation in winter. The studies conducted by Soliman et al. [7] and Gao et al. [9] suggest that employing sophisticated and comprehensive models that incorporate multiple variables can result in improved outcomes in climate change impact studies.

We are very grateful to MDPI journals for their trustworthy collaboration in developing this Special Issue, “Climate Change and Insects”. Working on this Special Issue gave us—the editors—the chance to be in contact with authors and reviewers all over the world and discuss their—sometimes quite different—opinions and conclusions about insects in a changing environment on a global scale. Despite opposite meanings, we are profoundly moved by the high number of potential effects described in the research studies leading to a general hypothesis: the fine equilibrium between insect species, their interactions, and their role in their habitats are highly endangered. In consequence, our Special Issue reflects that entomologists all over the world have identified significant impacts of CC inside the insect community worldwide. Especially in terms of plant protection aspects in agriculture, controlling insect pests and their role as vectors of plant pathogens will be a demanding challenge in this century [16]. In this Special Issue, we have collected scientific work to highlight potential risks for food production, species conservation, and the function of ecosystems. While we possess a comprehensive understanding of the potential implications for the insect world under future climate conditions, our level of preparedness remains significantly inadequate.
